# Supplementary material for: Leonurine inhibits cardiomyocyte pyroptosis to attenuate cardiac fibrosis via the TGF-β/Smad2 signalling pathway
Source: PLoS One. 2022 Nov 3;17(11):e0275258. doi: 10.1371/journal.pone.0275258 (PMC9632889; doi:10.1371/journal.pone.0275258)

Fig. 4A

FN1, COL3A1, α-SMA, and β-actin were run on the same gel, cut before incubation with antibodies, and detected using a GE Amersham Imager 800 RGB (EG, USA).

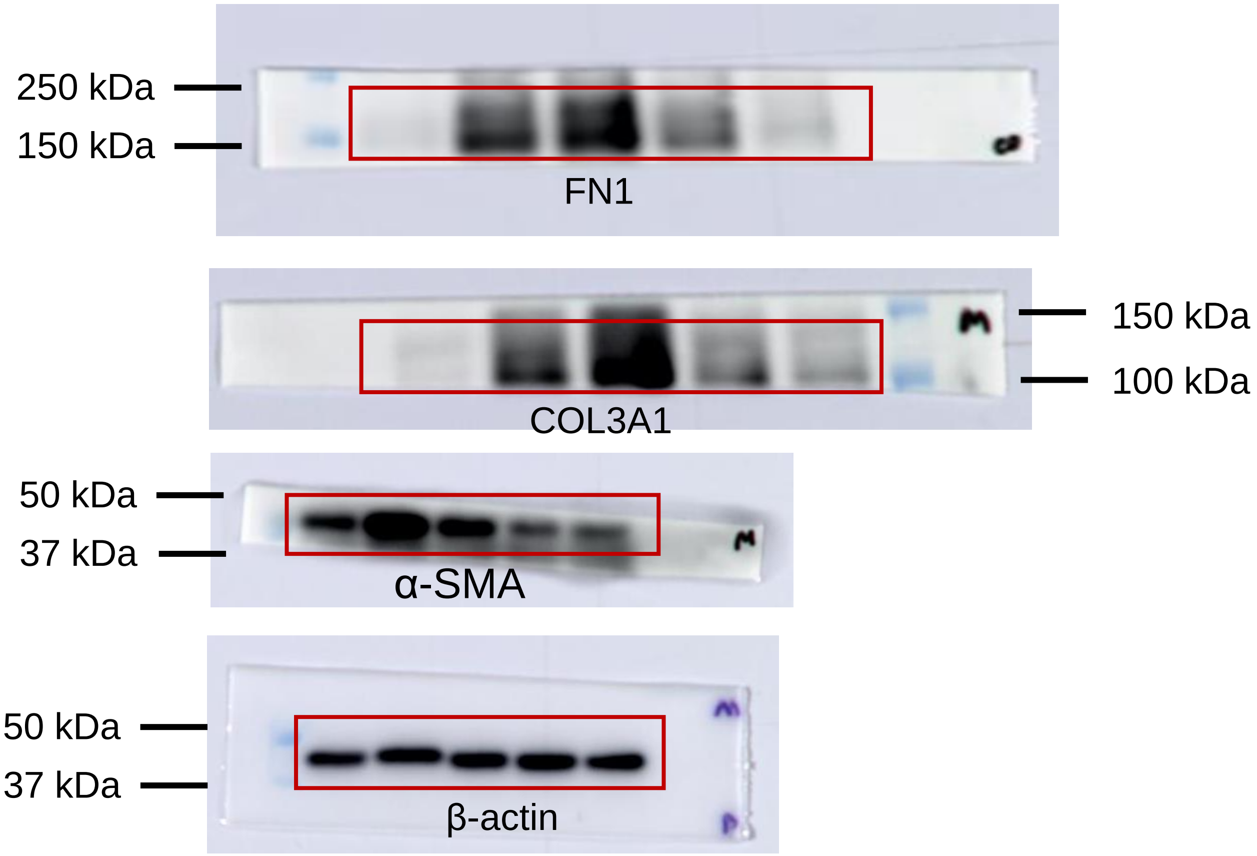

Fig. 5A

GSDMD, Caspase 1, Cleaved GSDMD, Cleaved Caspase 1, and β-actin were run on the same gel, cut before incubation with antibodies, and detected using a GE Amersham Imager 800 RGB (EG, USA).

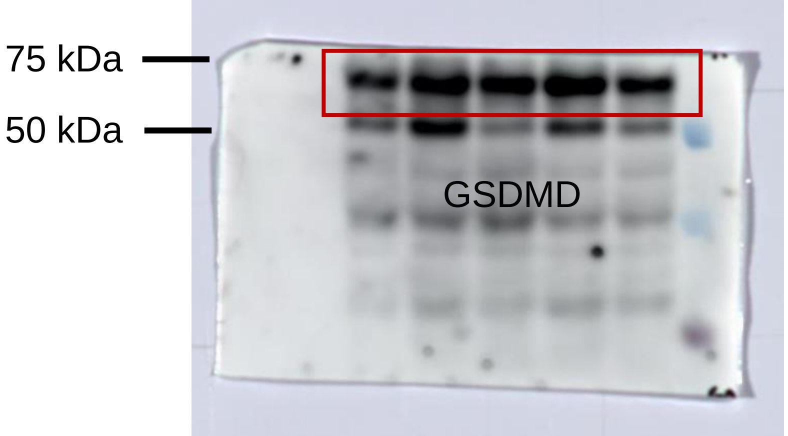

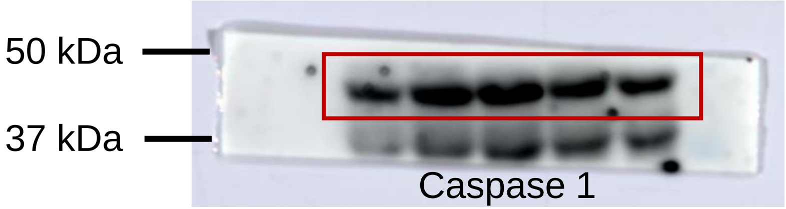

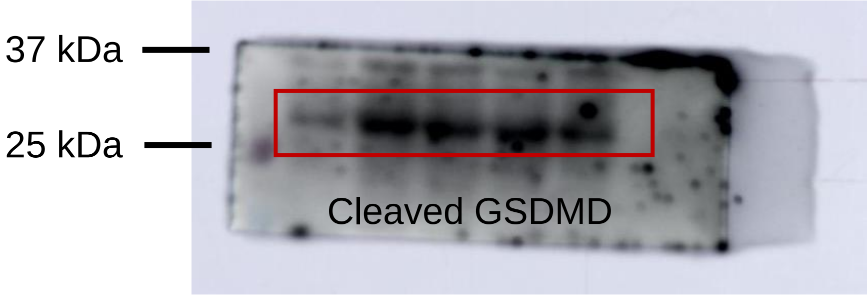

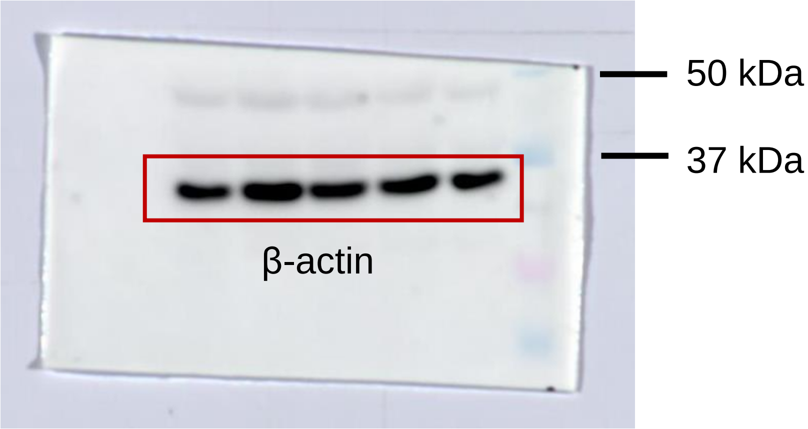

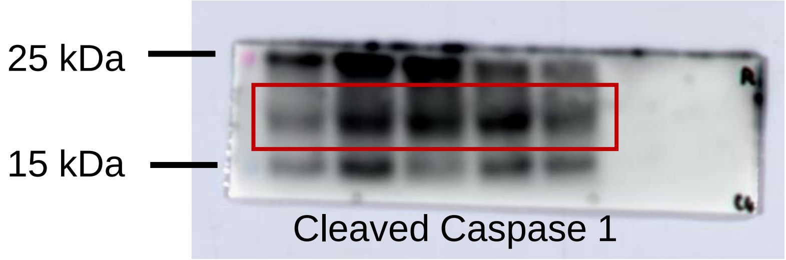

Fig. 6A

FN1, COL3A1, α-SMA, and β-actin were run on the same gel, cut before incubation with antibodies, and detected using a GE Amersham Imager 800 RGB (EG, USA).

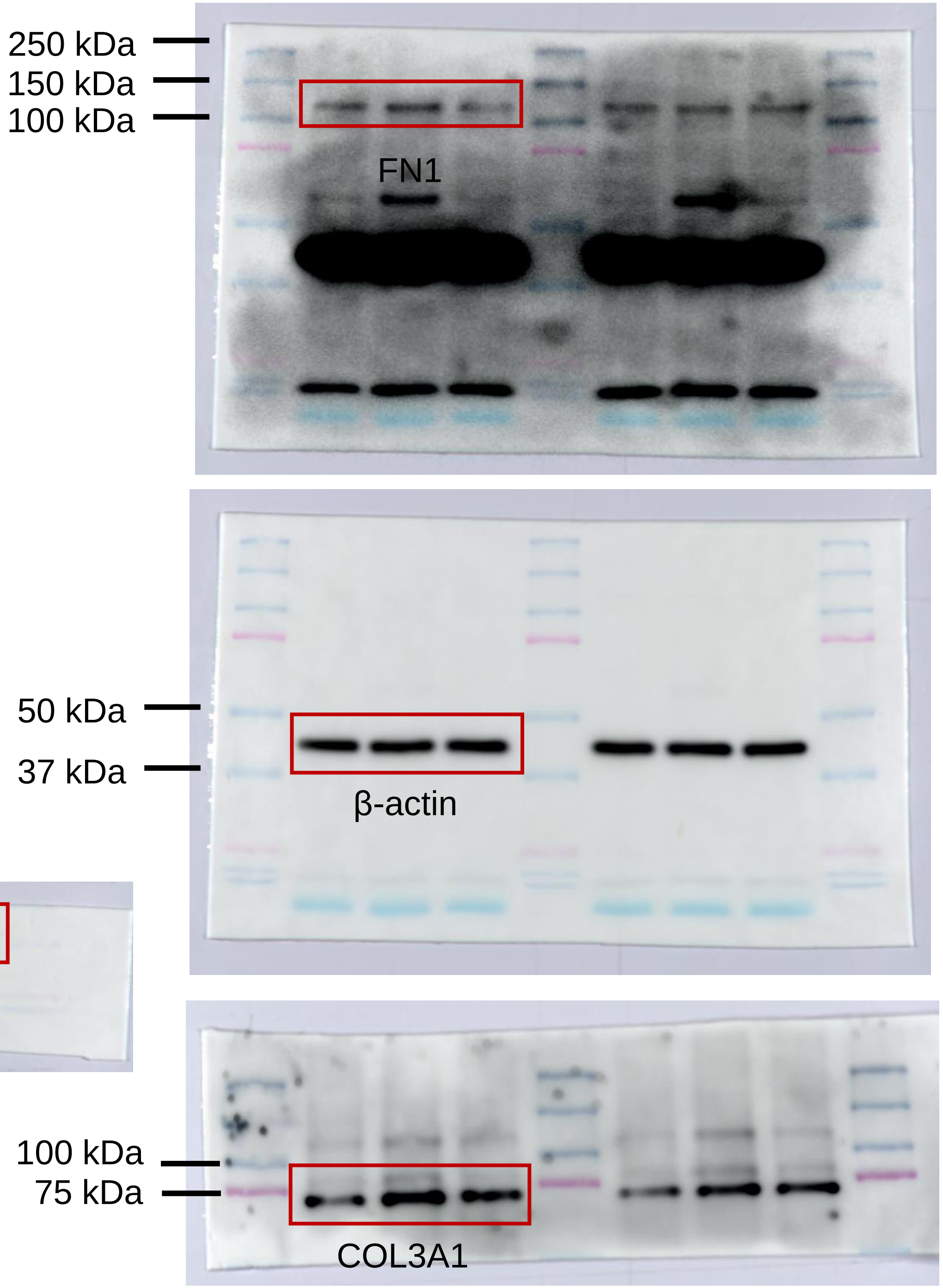

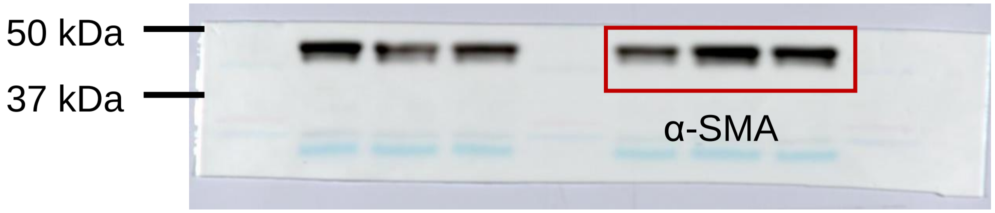

Fig. 7A

GSDMD, Caspase 1, Cleaved GSDMD, Cleaved Caspase 1, and β-actin were run on the same gel, cut before incubation with antibodies, and detected using a GE Amersham Imager 800 RGB (EG, USA).

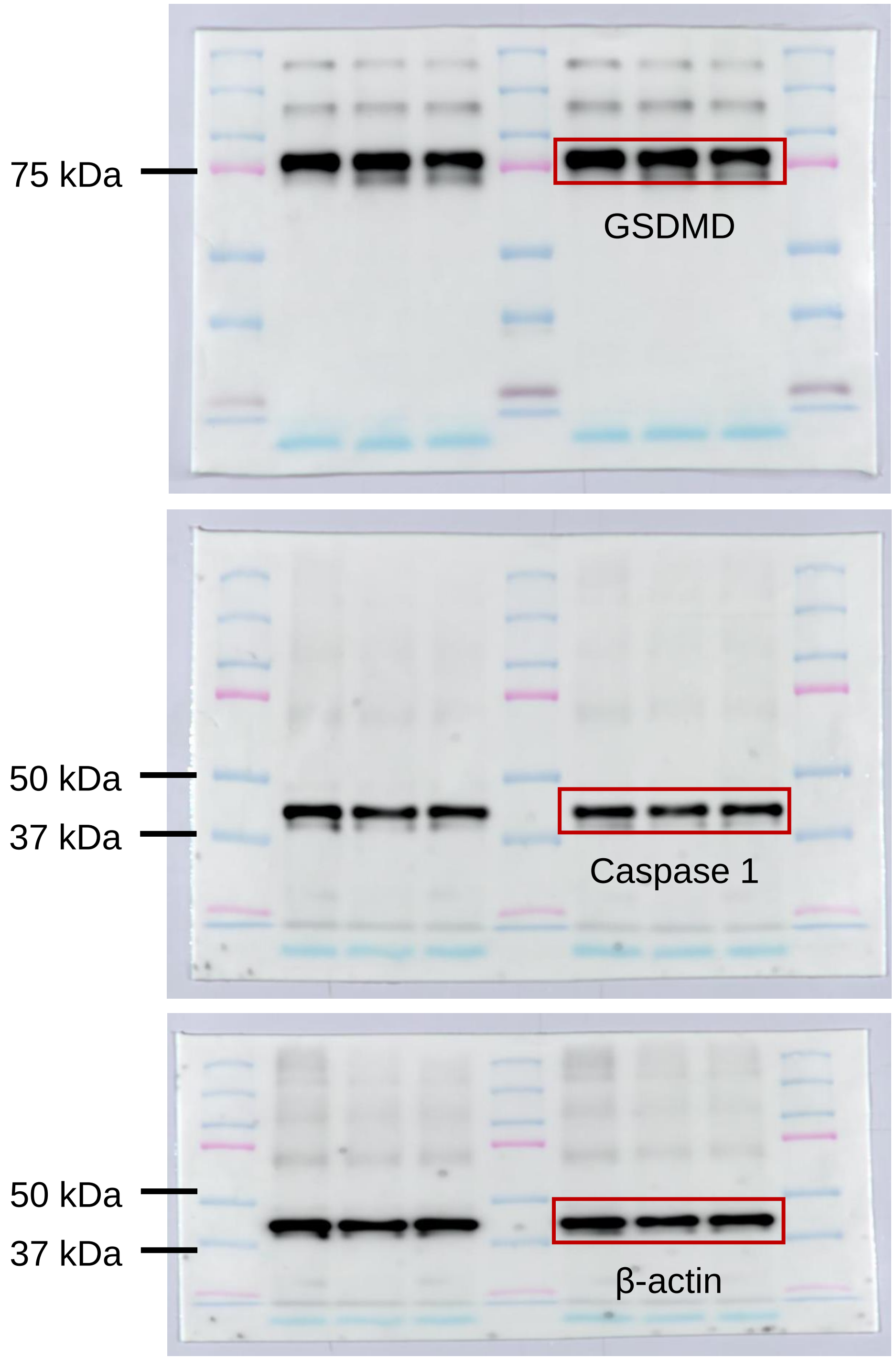

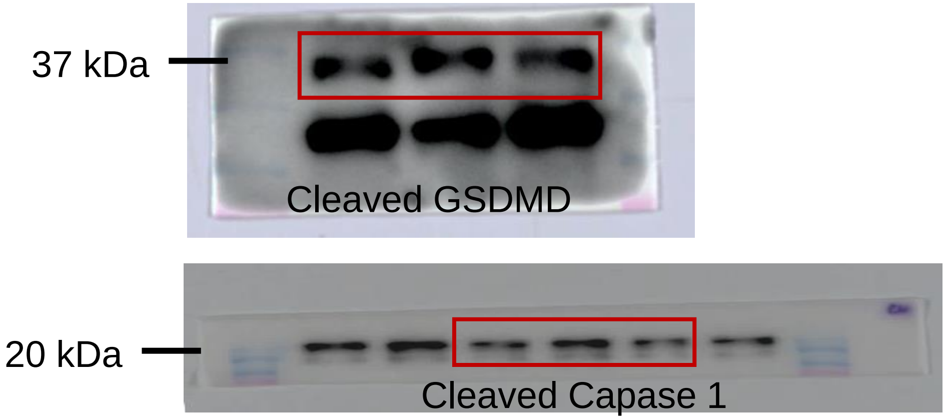

Fig. 9A

P-Smad2, Smad2, Caspase 1, Cleaved Caspase 1, and β-actin were run on the same gel, cut before incubation with antibodies, and detected using a GE Amersham Imager 800 RGB (EG, USA).

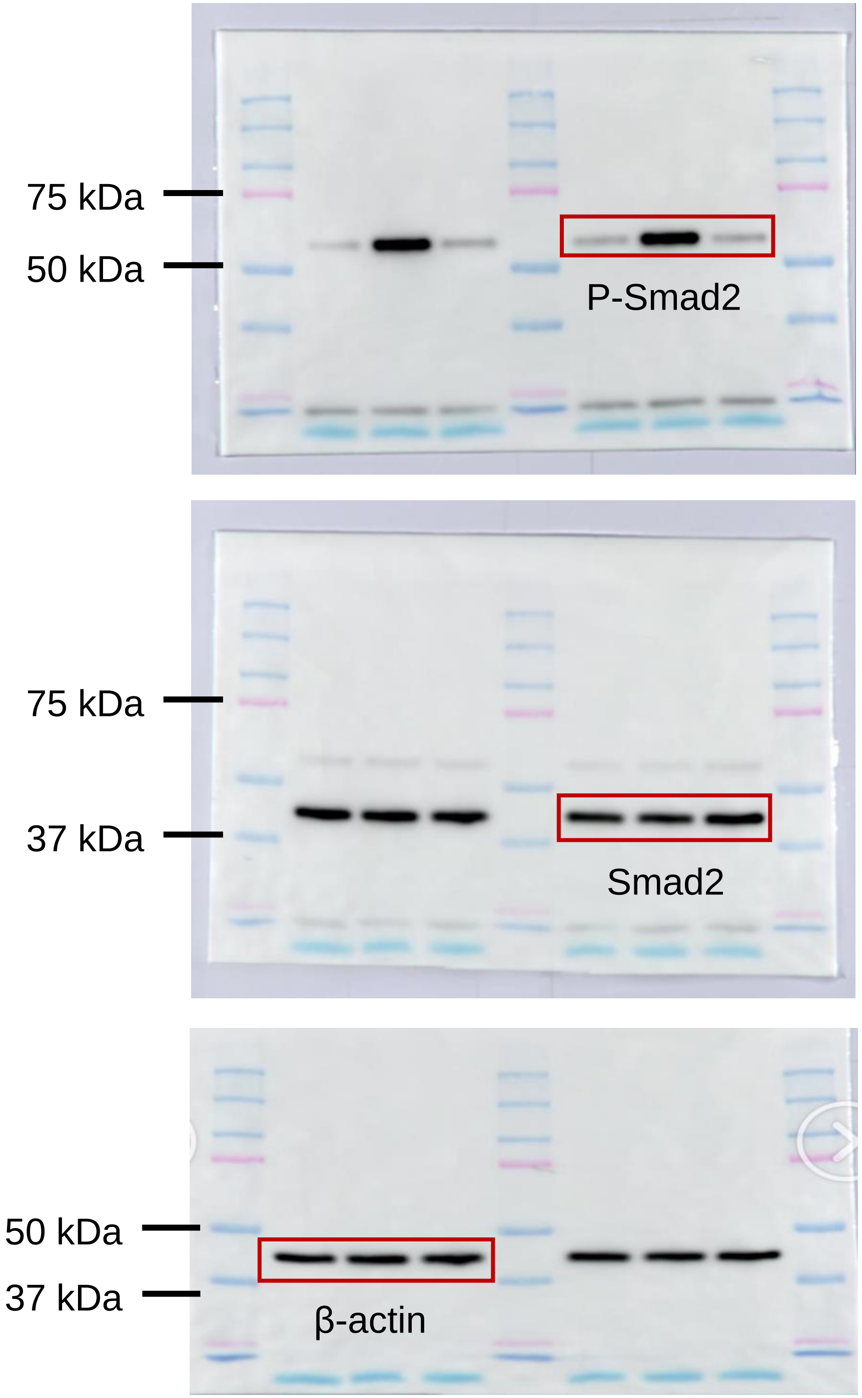

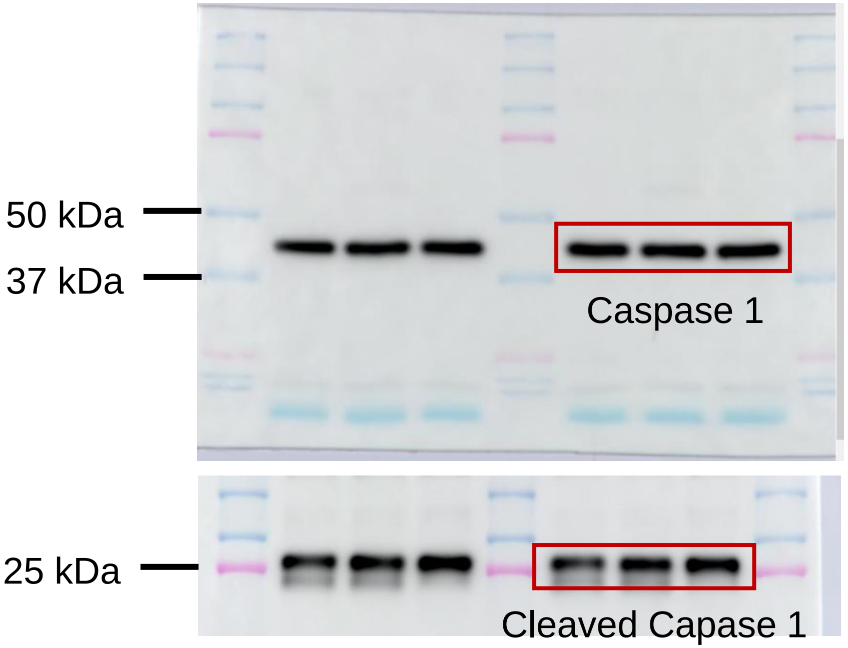

Supplement: S1 Raw images — (PDF) [file pone.0275258.s005.pdf]
