# Supplementary material for: Leonurine inhibits cardiomyocyte pyroptosis to attenuate cardiac fibrosis via the TGF-β/Smad2 signalling pathway
Source: PLoS One. 2022 Nov 3;17(11):e0275258. doi: 10.1371/journal.pone.0275258 (PMC9632889; doi:10.1371/journal.pone.0275258)
Supplement: Attachment — Submitted filename: Review PONE-D-22-05865 LSLM.pdf [file pone.0275258.s006.pdf]

Leonurine inhibits cardiomyocyte pyroptosis against cardiac fibrosis via the TGF-β/Smad2 signaling pathway
Reviewer Recommendation and Comments

I have had the pleasure of reading Li and colleagues' manuscript. The research manuscript aimed to answer the question whether the alkaloid Leonurine can block the progression of cardiac fibrosis and elucidate the molecular mechanism involving pyroptosis. I would like to suggest an English review by a native English-speaking reviewer for improve the understanding of the reader. Moreover, I further have a few comments on specific points in other to improve the manuscript understanding.

Major Concern:

1. Title: "Leonurine inhibits cardiomyocyte pyroptosis against cardiac fibrosis via the TGF-β/Smad2 signaling pathway." I think the word "against" leads to the reader a misunderstanding about the main view of the manuscript. I would suggest, "Leonurine inhibits cardiomyocyte pyroptosis via the TGF-β/Smad2 signaling pathway to prevent/block (the right word depends on the answer of question 4.c.) cardiac fibrosis.

2. Abstract: The citation (line 7): "*The manuscript studied the novel mechanism…*" please change for "This report…".

3. Introduction: It is missing the working hypothesis of the work. It is confusing from line 51 through the end of the paragraph. Reference 16 mentioned in the manuscript is also about the attenuation of myocardial fibrosis. Moreover, I don´t understand what the authors means that it has a great cardioprotective effect. Are the authors related this sentence with cardiac function (LEVDP, LVSP, Dp/Dt) preservation? If the answer is yes, what is the new point of this manuscript? Please clarify the working hypothesis enhancing the differences from previous publications.

4. Materials and Methods:
a. Leuronine was obtained from Fudan University; to me it seems that the substance is not commercially available. Is it as extraction? The authors should provide more information.

b. The authors should provide the total *n* number and mention if the treatment induced any loss of the animals.

c. Treatment protocol is confusing. To all rats, ISO was injected during 48 days. When exactly Leuronine was orally administrated? During ISO administration or after? For how long, 8 days? This may change the interpretation of the data. If the Leuronine was treated after ISO administration for 8 days, the substance reverts cardiac fibrosis. This issue should be discussed.

5. Results: The quality of Fig 3 is poor. The yellow mentioned in the text can´t be seen.

6. Discussion:
a. Paragraphs 1 and 2 says the same things, please be concise.

b. ISO is a beta-adrenergic agonist. Do the ISO-induced pyroposis mediated by beta-adrenergic receptor or is a consequence of the sustained augmentation of cardiac haemodinamic that may stimulate others mediators? If the pyroposis is mediated beta-adrenergic receptor, what is the difference of the treatments with beta adrenergic antagonist (such as atenolol) and Leuronine. Are co-treatment with both drugs synergic? The authors should provide the data or discuss this issue.

c. Leuronine is an alkaloid, if ISO was administered in concomitance to Leuronine, the physically interaction of both drugs is possible? Or, is Leuronine able to bind to beta adrenergic receptor? Another possibility is ROS chelation? These points as missed in the discussion.

d. At the end of the discussion, the authors mentioned “some limitations”, the authors should cite and discuss it better.

Minor Concern:

1. Please, provide the *p* values of each result in order for a better conclusion of the reader.
2. Please, revise Figure legends 1, 2, 3 and 4. The letters that label’s the panels are exchanged.´
3. It is encourage that the authors draw a picture underling the proposed molecular mechanism of Leuronide.
